# A study of quantum Berezinskii–Kosterlitz–Thouless transition for parity-time symmetric quantum criticality

**DOI:** 10.1038/s41598-021-84485-2

**Published:** 2021-03-09

**Authors:** Sujit Sarkar

**Affiliations:** 1 Department of Theoretical Sciences, Poornaprajna Institute of Scientific Research, 4, Sadashivanagar, Bangalore, 560 080 India; 2 Poornaprajna Institute of Scientific Research, Bidalur Post, Devanhalli, Bangalore Rural, Bangalore, 562110 India

**Keywords:** Quantum physics, Topological matter

## Abstract

The Berezinskii–Kosterlitz–Thouless (BKT) mechanism governs the critical behavior of a wide range of many-body systems. We show here that this phenomenon is not restricted to conventional many body system but also for the strongly correlated parity-time (PT) symmetry quantum criticality. We show explicitly behaviour of topological excitation for the real and imaginary part of the potential are different through the analysis of second order and third order renormalization group (RG). One of the most interesting feature that we observe from our study the presence of hidden QBKT and also conventional QBKT for the real part of the potential whereas there is no such evidence for the imaginary part of the potential. We also present the exact solution for the RG flow lines. We show explicitly how the physics of single field double frequencies sine-Gordon Hamiltonian effectively transform to the dual field double frequencies sine-Gordon Hamiltonian for a certain regime of parameter space. This is the first example in any quantum many body systems. We present the results of second order and third order RG flow results explicitly for the real and imaginary part of the potential. This PT symmetric system can be experimentally tested in ultra-cold atoms. This work provides a new perspective for the PT symmetric quantum criticality.

## Introduction

Symmetries are essential concept to understand and also to describe the physical system^1^. The laws of physics to be invariant under symmetries. The physics to be equivalent for charge conjugation symmetry (*C*), parity symmetry (*P*) and time reversal symmetry (*T*). The most interesting feature of symmetry breaking^2^ is that it creates the non-trivial and emergent physics of different quantum phases in quantum many body system.

PT symmetry has its roots in quantum field theory and opens a new perspective in studying non-Hermitian Hamiltonians. It can exhibit spontaneous symmetry breaking accompanied by a real-to-complex spectral phase transition. PT symmetric quantum mechanics is an extension of conventional quantum mechanics into complex domain^3–6^.

Initially PT symmetry quantum mechanics has started as an interesting mathematical discovery and a good theoretical exercise for theoretical physics^3–6^. But in current literature, it has been expanded experimentally in different field of science like open quantum systems^7,8^, physics of gain and loss (as found in photonics^9–11^) or systems where the non-Hermiticity models the finite lifetime^12,13^, localization–delocalization^14–16^, biological systems^17–19^, Weyl semi-metals^20–24^, topology and dissipation^25,26^ and PT symmetric circuit QED^27^.

This exceptional (EP) point is the unique feature in non-hermitian system when its complex eigen values and the corresponding eigenstate may coalesce, giving rise to a non-hermitian degeneracy^3–6,28–31^. This EP has a dramatic effect on the system leading to the nontrivial physics with interesting counterintuitive features, which we will observe in this study. These striking properties have been observed experimentally in microwave experiments^32–34^, nuclear magnetic resonance^35^, optical system^36^.

In the present study, we consider the low energy excitations of one-dimensional quantum many body system. This system shows only the collective excitations. The Tomonoga–Luttinger physics provide a unified description to study the low-energy behaviour for this system. This collective excitations of the system can be described in terms of bosonic field and also finally to express the model Hamiltonian of system in sine-Gordon model Hamiltonian. The study of this sine-Gordon model Hamiltonian gives different interesting features of quantum criticality^37–39^. One can use renormalization group^40,41^ method to extract the different quantum phases from the analysis of sine-Gordon Hamiltonian.

### Motivation

The Berezinskii–Kosterlitz–Thouless (BKT) mechanism governs the critical behavior of a wide range of many body system. The main motivation of this study is to show that this phenomenon is not restricted to conventional many body system but also for the strongly correlated parity-time (PT) symmetric quantum criticality.

At the BKT transition temperature the pairs unbind and the vortices proliferate, resulting in a state with no spin rigidity and the correlation function decays exponentially with temperature. This is the classical picture of the BKT transition^42–45^. In quantum BKT (QBKT), there are no temperature dependent transitions as it observed for the classical BKT. For this case topological transition depends on the strength of sine-Gordon coupling and the Luttinger liquid parameter (*K*). QBKT transition is a different type of transition, where there is no spontaneous symmetry breaking whereas spontaneous symmetry occurs in the PT symmetry breaking phase excitations.

This model Hamiltonian is the single field double frequencies sine-Gordon model Hamiltonian. We will see how the nature of the model Hamiltonian changes for the different phases of the PT symmetric quantum criticality. This work provides a new perspective for quantum criticality in quantum many body systems. We use renormalization group (RG) method to solve this QBKT problem for PT symmetry quantum criticality. The mathematical structure and results of the RG theory are a significant conceptual advancement in quantum field theory in the last several decades in both high-energy and condensed matter physics^46,47^. We use six sets of RG equations which cover the second and third order RG process to solve the present problem.

### The model Hamiltonian and renormalization group equation

We consider a class of one-dimensional quantum system described by the sine-Gordon field theory

1 $$\begin{aligned} H = H_{0} + V( \phi ), \end{aligned}$$

where $$H_{0}$$ is

2 $$\begin{aligned} H_0 = \left( \frac{{h} v}{2 \pi }\right) \int dx \left[ K {({\partial _x \theta (x)})}^2 + \frac{1}{K} {({\partial _x \phi (x)})}^2 \right] . \end{aligned}$$

The Hamiltonian, $H_0$ gives a universal framework for describing one dimensional interacting bosons and fermionic system, i.e., Tomonoga–Luttinger liquid (TLL) Hamiltonian and $V (\phi )$ is the sine-Gordon potential. $\theta (x)$ is the dual field of $\phi (x)$ and satisfy the following commutation relation , $[ \phi (x), \partial _x \theta (x') ] = -i \pi \delta (x - x')$. *v* is the velocity of the collective excitation of the system, *K* is the Tomonoga–Luttinger liquid (TLL) parameter to present the interaction strength in the system. The physics of low-dimensional quantum many body condensed matter system is enriched with its new and interesting emergent behavior. $K < 1$ and $K > 1$ and $K=1$ characterizes the repulsive, attractive interactions and non-interacting, respectively^37–39^.

In the correlated many-body system, this situation is described by the celebrated sine-Gordon model, which also plays a central role in quantum field theory. However, a richer and more difficult to understand class of topological transitions for sin-Gordon model Hamiltonian which play a major role for several systems^37–39^. A generalization to the PT symmetric case by adding an imaginary contribution^29^ to the potential term is as follows.

3 $$\begin{aligned} V (\phi ) =\frac{\alpha _r}{\pi } cos (2 \phi ) - \frac{i \alpha _i }{\pi } sin (2 \phi ), \end{aligned}$$

where $\alpha _r$ and $\alpha _i$ are the real and imaginary part of the potential. The imaginary part of the potential, which introduces physics of spectral singularity occurs when the real and imaginary part of the potential are same^29^.

It will observe that when $\alpha _r$ becomes relevant, a stable gapped phase , i.e, the fluctuation of $\phi$ gets suppressed. But when $\alpha _i$ becomes relevant, it facilities the fluctuations of $\phi$. Therefore finally it behaves like an effectively dual field sine-Gordon model Hamiltonian. The phase diagram is thus controlled by the confinement/deconfinement of the corresponding dual topological excitations. These situations are considerably more difficult to analyze and need much more sophisticated field theory descriptions such as the so-called dual-field double sine-Gordon model^46^.

This model Hamiltonian (Eq. 1) has enrich physics. In the threshold of the PT transition this Hamiltonian system has spectral singularity and quantum criticality conspire to yield an unconventional RG fixed point^29^, which has no counterpart in Hermitian system. The other important and interesting physics is due to the presence of imaginary potential, at the relevant phase of this there is local gain and loss physics triggers an enhancement of superfluid correlation.

To get the correct physical picture of quantum criticality for this model Hamiltonian, RG study is essential^35–37^. Here we present the second (2nd) order and also third (3rd) order RG equations. Detailed derivations of bosonized Hamiltonian and RG equations are relegated to “Methods” section. The analytical expressions for the 2nd order RG are the following:

4 $$\begin{aligned} \frac{d g_r }{dl}&= \left( 2-K \right) g_r \nonumber \\ \frac{d g_i }{dl}&= \left( 2-K \right) g_i \nonumber \\ \frac{dK}{dl}&= ({g_i}^2 - {g_r}^2 ) K^2 . \end{aligned}$$

The analytical expressions for the 3rd order RG are the following:

5 $$\begin{aligned} \frac{d g_r }{dl}&= \left( 2-K \right) g_r + 5 {g_r}^3 - 5 {g_i}^2 g_r \nonumber \\ \frac{d g_i }{dl}&= \left( 2-K \right) g_i - 5 {g_i}^3 + 5 {g_r}^2 g_i \nonumber \\ \frac{dK}{dl}&= ({g_i}^2 - {g_r}^2) K^2 . \end{aligned}$$

Here *l* is the logarithmic RG scale and $g_{r,i} = \frac{\alpha _{r,i} a^2}{h v}$ are the dimensionless coupling constants with *a* being a short distance cut-off and *v* is the collective excitations. Here these equations describe as the whole set of RG equations because both the couplings of the Hamiltonian, i.e., $g_r$ and $g_i$ are present.

**Figure 1:**
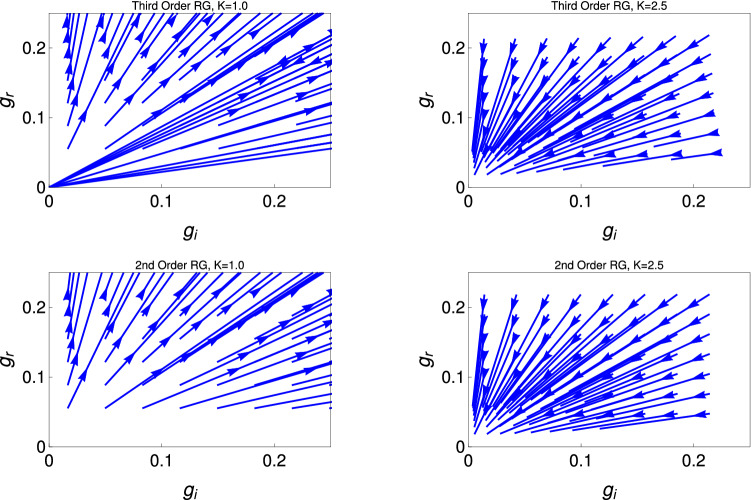
These figures show renormalization group flow diagram for the couplings $g_r$ with $g_i$, for two different initial values of $K (= 1, 2.5)$. The upper and lower panels are for the third order (Eq. 5) and second order (Eq. 4) RG equations solutions, respectively.

**Figure 2:**
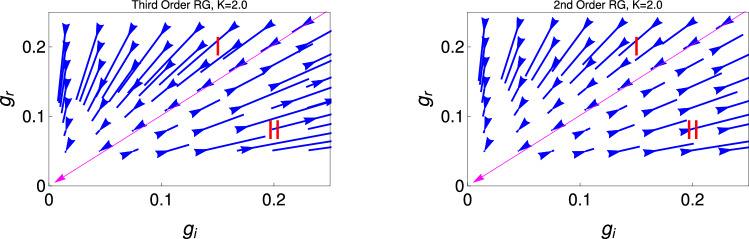
These figures show the behaviour of renormalization group flow lines for the couplings $g_r$ and $g_i$, for the initial value of $K (=2)$. The left and right figures are for the third order (Eq. 5) and second order (Eq. 4) RG equations solutions. We mark the regions of RG flow lines into two region, I and II. The region I and II are the PT symmetry preserve and broken phases respectively. Magenta colour with arrow is the line of separatrix between the PT symmetry and PT symmetry broken phase. The BKT and PT phase boundaries coincide at $K=2$.

## Results along with physical interpretations

### Whole sets renormalization group equations and effective single frequency model Hamiltonian

Figure 1 represent the RG flow diagram for the couplings $g_r$ and $g_i$. It consists of two panels to present the results of two different order RG studies. The upper and lower panel are respectively for the third order (Eq. 5) and second order (Eq. 4) RG study. Each panel consists of two figures, which shows behaviour of RG flow lines for lines for the couplings $g_r$ with $g_i$, for different initial values of *K*(0). The left and right figure in each panel are for $K=1$ and 2.5, respectively. It reveals from this study for the smaller initial values of $K (=1.0)$, both the real ($g_r$) and imaginary potential ($g_i$) flowing off to the strong coupling phase.

For the right figures of each panel, $K=2.5$, RG flow lines for both the couplings ($g_r$ and $g_i$) are flowing off to zero. In this phase, the system is in gapless TLL phase.

**Figure 3:**
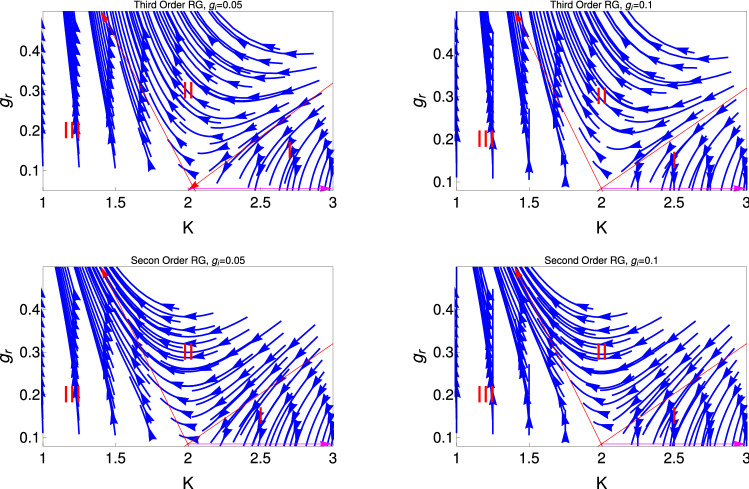
These figures show behaviour of RG flow lines, $g_r$ with *K* two different initial values $g_i (0)$. The upper (Eq. 5) and lower (Eq. 4) panels are, respectively, for results of third and second order RG studies. We mark different regions of quantum phases by I, II, III and the separatrix by the red colour lines. The magenta colour line represent the fixed line.

Figure 2 represent for $K=2$, there is a transition between PT symmetry state to the PT symmetry broken state. We present the behaviour of RG flow lines for the different initial for the couplings ($g_r$ and $g_i$ ) but with the fixed initial values of $K=2$. It reveals from this study that the region (I) ($g_r (0) > g_i (0)$) is the PT symmetric phase region and region (II) is the PT symmetric broken phase region. These two regions are separated by the separatrix (magenta line with arrow towards to the origin). We will see from the RG Eqs. (7)–(10) , and also Fig. 3, that $K=2$ is the QBKT transition point. It reveals from this study that at $K=2$, the model Hamiltonian also show the PT symmetry transition thus $K=2$ has some special important feature where the QBKT transition and PT symmetry phase boundaries coincides. The line which separated the PT symmetry and PT broken phase ($g_r = g_i$), which term as spectral singular critical point.

There is some difference between the spontaneous symmetry breaking of PT symmetry and other quantum many body systems like superconductivity, magnetism etc. In this spontaneous symmetry breaking phase the symmetry of the system reduced. According to the Mermin–Wigner–Ho theorem there is no spontaneous symmetry breaking low dimensional system ($d \le 2$)^41^. But the PT symmetry is spontaneously broken in eigenstates. PT symmetry is said to be broken if some of the eigenstate of the Hamiltonian are not the eigenstate of the PT operators even though $[H, PT]= 0$. In this phase, the system is in gapless TLL phase. Therefore it is found from this figure that only $K =2$, the system shows the transition from PT symmetry state to PT symmetry broken state.

On the PT symmetry threshold line the spectral singularity arises as well as their eigenstates coalesce in the continuum spectrum. Finally it become clear that in quantum many body system coexistence of the spectral singularity and quantum criticality can results in an exotic RG fixed point unique to non-Hermitian point.

Figure 3 shows the RG flow lines for the couling $g_r$ with *K* for two different initial values of $g_i (0)$ (Eqs. 4, 5). This figure consists of two panels, where upper and lower panels are respectively for the 3rd order and 2nd order results. It reveals from this study that this RG flow diagram has three different region of phases, one is weak coupling phase (region mark by I), i.e., the system is in TLL phase for $K >2$ and another region is the strong coupling phase, i.e., the gapped phase for $K < 2$ . In this strong coupling (region mark by III) phase, the system is in the Mott insulating (MI) phase. Apart from that there is the crossover phase (region mark by II) of the system which goes from the weak coupling TLL phase to the strong coupling MI phase. It reveals from this study of this figure that crossover regime is more prominent for the 3rd order RG study. We have observed from the study of Fig. 3 that the behaviour of RG flow lines shows the hidden QBKT behaviour, i.e, RG flow lines show three different regime as one observed for the conventional BKT RG flow diagram although this RG equation is not the conventional QBKT. Therefore this behaviour of the RG flow diagram is termed as hidden QBKT.

In one-dimensional quantum many body bosonic system subject to periodic potential, the locking favours the suppression of density fluctuations and thus the gapped phase corresponds to the Mott-insulator phase (MI). This is physical picture for QBKT phase transition, one can also interpret this physical picture interms of classical picture as condensation of bounded pairs of vortex and anti-vortex.

In the weak coupling regime, $K \ge 2$, the effect of perturbation appears as a renormalization of the value of *K* and $g_r$. It is found from the flow diagram that $g_r$ effectively decreases with *K* and effectively ending up at the renormalized value of $K (= K^{\star })$. The base line, $K \ge 2$ (magenta colour line) is the fixed line for this RG flow diagram.

#### Effective single frequency sine-Gordan Hamiltonian

Now we explain the physics for the PT symmetry unbroken phase. When $\alpha _r > \alpha _i$ the spectrum is real and the PT symmetry is preserve $^{29}$. The author of Ref.^48^ has shown explicitly that the spectrum is real if and only if there exist an operator $\hat{O}$ satisfying ${\hat{O}}^{-1} H {\hat{O}} = \hat{H_0}$, $H_0$ is the Hermitian operator. He has constructed a operator such that $\hat{O}= e^{- \eta \frac{\theta _0 }{2} }$, where $\hat{{\theta }_0}$ is a constant part of $\hat{\theta }$ and $\eta = arctanh( {\alpha _i}/{\alpha _r } )$. For this situation the interaction term become a single effective sine-Gordon Hamiltonian.

6 $$\begin{aligned} H_1 = (\frac{{h} v}{2 \pi }) \int dx [ K {({\partial _x \theta (x)})}^2 + \frac{1}{K} {({\partial _x \phi (x)})}^2 ] + \sqrt{ {\alpha _r}^2 - {\alpha _i}^2 }/{\pi } \int dx cos (2 \phi ) . \end{aligned}$$

Thus finaly, we find effective single frequency sine-Gordon Hamiltonian for the PT symmetric phase, even though the complex potential is present. There is no single frequency sine-Gordon model for the PT symmetry broken phase, because both the couplings ($g_r$ and $g_i$ ) increases with *K* for the smaller values of *K* and $g_i$ touches the base line (fixed line, magenta colour line of Fig. 3) for the higher values of *K*. Thus the single frequency sine-Gordon Hamiltonian is only for the PT symmetry preserving phase.

**Figure 4:**
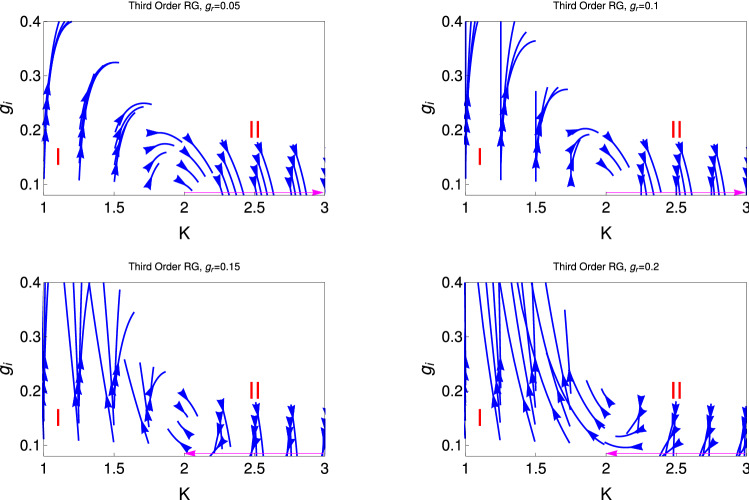
These figures show behaviour of RG flow lines, $g_i$ with *K* for four different initial values of $g_r (0) (0.05, 0.1, 0.15, 0.2)$ for the 3rd order RG process. The upper and the lower panels are respectively for the lower and higher values of $g_r (0)$. We mark different regions of quantum phases by I, II. The magenta colour line represents the fixed line. Region I and II are respectively the superfluid and TLL phase.

Figure 4 shows the behaviour of RG flow lines $g_i$ with *K* for four different initial values of $g_r (0) (= 0.05, 0.1, 0.15, 0.2)$ for the third order RG process. It reveals from this study that the behaviour of the RG flow lines are behave differently as we increase the initial values of $g_r (0)$. It is found that for the smaller initial values of $g_r (0)$ (upper panel of the figure) that the behaviour of the RG flow lines are behave as a convex character as we increase the initial values of $g_r (0)$, it transform to the concave character of the RG flow lines as we present in the lower panel of this figure. It is found that for the smaller initial values of $g_r (0)$ (upper panel of the figure), the RG flow lines are flowing off to the strong coupling phase upto the initial value of $K(0) =1.5$. We mark the region of strong coupling phase as I. The other phase of this system is the weak coupling phase, when the coupling $g_i$ touches the base line (fixed line, magenta colour line of the figure). i.e., the TLL phase which starts from $K =1.5$ and the second one is the strong coupling phase, i.e., the superfluid phase for $K<1.5$. In the lower panel, we present the behaviour of RG flow lines for the higher initial values of $g_r (0) (=0.15, 0.2)$, it is found that the character of the RG flow lines become concave and the region of superfluidity become large, it is $K(0) = 1.8$ and 2.2 for the two initial values $g_r = 0.15$ and 2 respectively. The concave character is more pronounced for $g_r (0) =0.2$.

**Figure 5:**
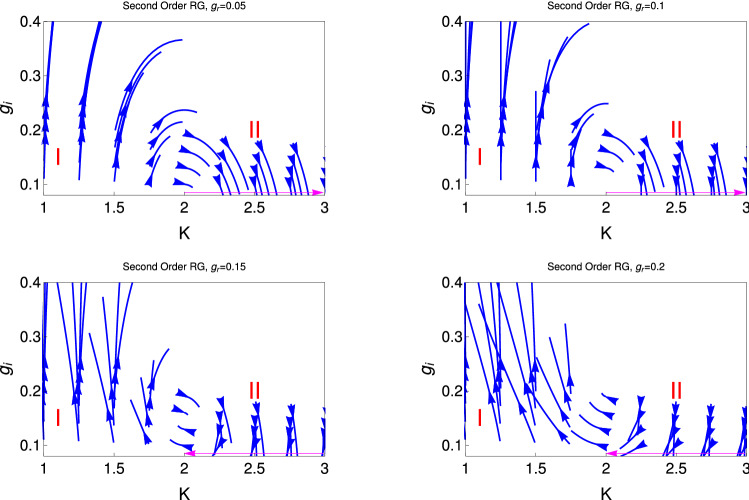
These figures show behaviour of RG flow lines, $g_i$ with *K* for four different initial values of $g_r (0) (0.05, 0.1, 0.15, 0.2)$ for the 2nd order RG process. The upper and the lower panels are respectively for the lower and higher values of $g_r (0)$. We mark different regions of quantum phases by I, II. The magenta colour line represents the fixed line. Region I and II are respectively the superfluid and TLL phase.

In Fig. 5, we do the same study what we have done for the Fig. 4 but for the 2nd order RG equations. We observe the same behaviour and the transition point from the superfluid state to the TLL phase occur as one observe for the 3rd order RG. But for the 2nd order RG process, we observe that the transition point for the superfluid to TLL phase changes , now it is $K[0] = 1.7$ and 2 respectively. Thus it is clear from this study that the strong coupling regime is more prominent for the 3rd order RG study.

### Quantum Berezinskii–Kosterlitz–Thouless transition along with physical interpretation

Here we derive four sets of conventional QBKT equations from the 2nd order (Eq. 4) and 3rd RG (Eq. 5) equations (Please see/// “Method” section for the detail derivation). In the previous section we have already discussed the basic aspects of QBKT and also the motivation of the present study.

7 $$\begin{aligned} \frac{d g_r }{dl}&= \left( 2-K \right) g_r \nonumber \\ \frac{dK}{dl}&= - {g_r}^2 K^2 . \end{aligned}$$

8 $$\begin{aligned} \frac{d g_i }{dl}&= \left( 2-K \right) g_i \nonumber \\ \frac{dK}{dl}&= {g_i}^2 K^2 . \end{aligned}$$

Here we also derive two sets of quantum BKT equations from the third order RG equations

9 $$\begin{aligned} \frac{d g_r }{dl}&= \left( 2-K \right) g_r + 5 {g_r}^3 \nonumber \\ \frac{dK}{dl}&= - {g_r}^2 K^2 . \end{aligned}$$

10 $$\begin{aligned} \frac{d g_i }{dl}&= \left( 2-K \right) g_i - 5 {g_i}^3 \nonumber \\ \frac{dK}{dl}&= {g_i}^2 K^2 . \end{aligned}$$

**Figure 6:**
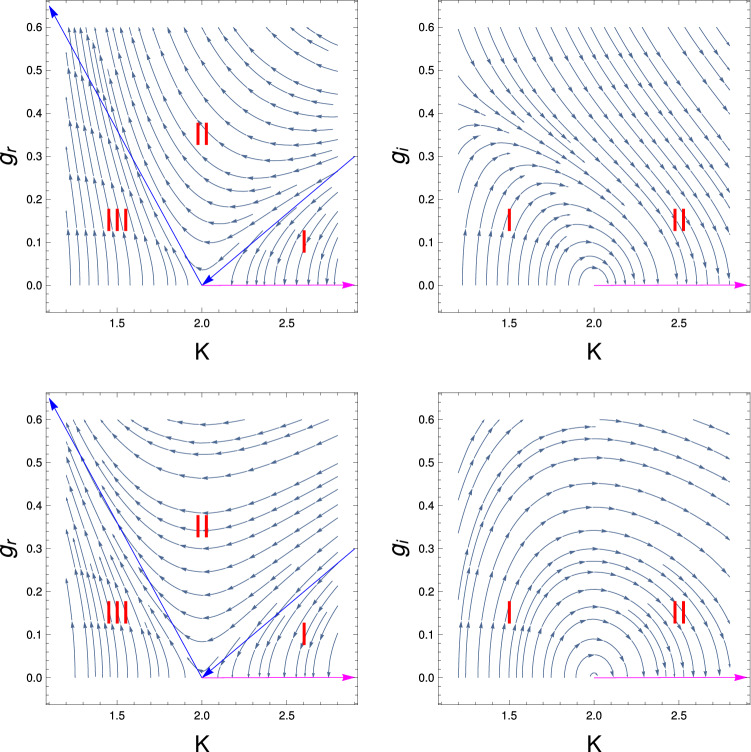
These figures show the behaviour of RG flow lines for the couplings $g_r$ and $g_i$ to study the conventional quantum BKT equations (Eqs. 7, 8, 9). The upper and lower panels are respectively for third order and second order RG study. We mark different regions of quantum phases by I, II, III and the separatrix by the red colour lines. The magenta colour line represent the fixed line. RG flow lines show different behaviour for the couplings $g_r$ and $g_i$ and also for the 2nd and 3rd order RG process.

Figure 6 represents results of QBKT study based on four sets of RG equations (Eqs. 7, 8, 9, 10). The upper and lower panels are respectively present results of third-order and second-order RG calculations. Each panel consists of two figures, the left and right figure are respectively for the RG flow study of $g_r$ and $g_i$ with *K*. It is found that the left figures of upper and lower panels have three regions, weak coupling region (mark by I in the figure) where the RG flow lines for $g_r$ flowing off to the zero, i.e, the system is in the gapless TLL phase, upto $K=2$. In the strong coupling region (mark by III in the figure), the RG flow lines for the coupling $g_r$ flowing off to the strong coupling phase and this phase system is in the gapped insulating phase. The other region is the cross over region, in this region system goes from weak coupling phase to the strong coupling phase (mark by II in the figure). The separatrix of different quantum phases are marked by the red colour lines and the fixed line by the magenta colour in the RG flow diagram. It is found that the results of 3rd order RG, the flow lines are much more stiffer compare to the 2nd order RG due to the presence of an extra term ($\sim {g_r}^3$ ) in the RG flow equations.

It is found from this study that the RG flow lines are more stiffer for conventional QBKT equation compare to RG flow lines of hidden BKT. We also observe the 2nd order RG flow lines for the coupling $g_i$ show only the semicircular nature for the all initial values of $g_i$. But for the 3rd order RG flow lines, it shows the semicircular nature for the smaller initial values of $g_i$, otherwise there is no evidence of semicircular nature of the RG flow lines.

The situation is different for the QBKT study for $g_i$ with *K*. Here we observe semicircular RG flow lines. The imaginary potential allows for substantial increase of K even if its strength $g_i$ initially small indicating the anomalous enhancement of the superfluid correlation. One can understand the anomalous enhancement of superfluid correlation from this physical interpretation: a local gain–loss structure introduced by the imaginary term causes locally equilibrated flows^29,49,50^ in the ground state. This results in the enhancement of fluctuations in the density, or equivalently, the suppression of fluctuations in the conjugate phase. For $K > 2$, both of the couplings are flowing off to the base line ($g_{r,i} =0$), i.e., the system is in the TLL phase.

It is found that in third order for the higher initial values of $g_i$ the RG flow lines are not semicircular in nature.

In one-dimensional quantum many body bosonic system subject to periodic potential, the locking favours the suppression of density fluctuations and thus the gapped phase corresponds to the Mott-insulator phase (MI). This is physical picture for QBKT phase transition, one can also interpret this physical picture interms of classical picture as condensation of bounded pairs of vortex and anti-vortex.

#### Nature of topological excitations and uniqueness of PT symmetry criticality

It is well known to us that the polifercation of topological excitations occurs in BKT transitions. But in the PT symmetry broken phase the ground state exhibits the enhanced superfluid correlation indicating tight binding of topological excitations^29,49,50^. Thus it is clear from these two topological excitations that these two transition behave different in nature.

The author of Refs.^37,38^ have solved the double frequencies dual field sine-Gordon Hamiltonian from the perspective of interacting Helical liquid and also for the study of topological states of interacting quantum matter. The model Hamiltonian of system contains two strongly relevant and mutually nonlocal perturbations over the Gaussian (critical) theory. In such a situation, the strong coupling fixed point is usually determined by the most relevant perturbation whose amplitude grows up according to its Gaussian scaling dimensions. However, this is not the general rule if the two operators exclude each other. In this case, the interplay between the two competing relevant operators, can produce a novel quantum phase transition.

The most interesting features of the PT symmetry quantum criticality the two couplings ($g_r$ and $g_i$) are present but there is only single field $\phi (x)$. But when the PT symmetry is broken, the fluctuation of the $\phi (x)$ facilitate the correlation of conjugate field. Therefore PT symmetry broken phase generate the physics of sine-Gordon dual field theory for the system. This is the uniqueness of PT symmetry quantum criticality.

Now we interpret the semicircular behaviour of RG flow lines physically, a real potential suppress the fluctuation of $\phi$ and stabilize the gapped Mott insulating phase for $K <2$. Moreover, owing to the semicircular RG flows, the imaginary potential allows for a substantial increase of *K*, even for the initial small values of $g_i$. This anomalous behaviour of our model Hamiltonian for the PT broken phase is also observed in the laser physics such as anomalous lasing and absorption in optics^29,49,50^.

To the best of our knowledge, it is the only phenomena in correlated many body system where the initial intuition was the real and imaginary potential are rival to the each other, as if they behave like dual fields to each other. But for the anamolous phase they are the true friend of each other.

**Figure 7:**
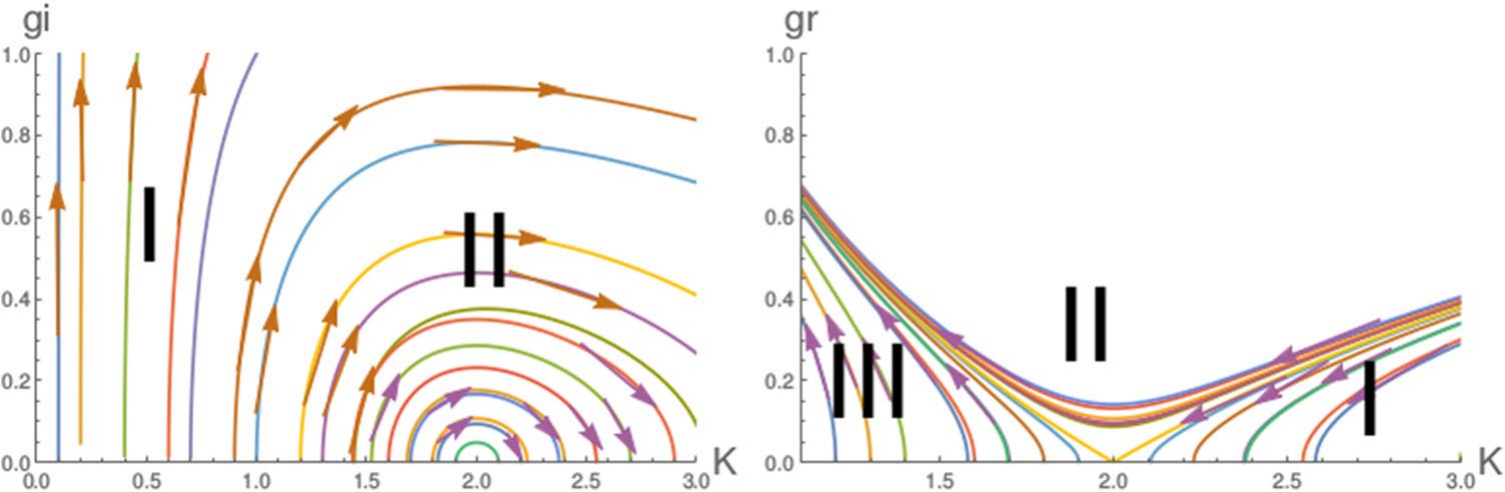
These figures show the RG flow lines for the exact solutions (Eqs. 11, 12) for the coupling $g_i$ (left figure) and $g_r$ (right figure). The regions mark by I and II for the left figures are respectively for the superfluid and TLL phase region. The regions mark by I, II and III for the right figures are respectively for the TLL, cross over phase and and strong coupling Mott insulating phase. Different colours for the RG flow lines have no extra physical significance.

#### Exact solutions

In this study, we exactly solve, two sets RG equations for the second order RG equations to find the behaviour of RG flow lines.

11 $$\begin{aligned} g_r= & {} \sqrt{ {{g_r} (0)}^2 + 4 (1/K -1/{ K_0} ) +2 ln(K/K_{0} ) }. \end{aligned}$$

12 $$\begin{aligned} g_i= & {} \sqrt{ {{g_i} (0)}^2 - 4 (1/K -1/{ K_0} ) - 2 ln(K/K_{0} ) }. \end{aligned}$$

In Fig. 7, we present the result of exact solutions of RG flow lines behaviour for real (right figure) and imaginary (left figure) potential. It is found that the behaviour of RG flow lines from the exact solutions (Eqs. 11, 12) are the same what we observe from the numerical studies of Eqs. (7) and  (8). The only difference that we obtain in the superfluid transition, for the exact solution it occurs at $K=1$, whereas we obtain it from the whole sets of RG equation (Eq. 4) at $K=1.5$.

It is clear from the results of exact solutions that the results of numerical studies are consistent with the exact solutions.

#### Experimental proposal

The authors of Ref.^29^ have proposed the source of non-Hermitian quantum mechanical system as PT symmetry quantum many body system. Here we briefly discuss their proposal. The PT symmetric many body system can be realized in a one-dimensional interacting ultracold bosonic atoms subject to a shallow PT symmetric optical lattice, the potential form is given in Eq. (3). $\alpha _r$ and $\alpha _i$ are respectively the depth of real and imaginary part of the potential, *d* is the lattice constant. The source of imaginary optical potential is a weak near-resonant standing-wave light. We refer, Ref.^29^, for the detail experimental proposal for this model Hamiltonian system.

## Discussion

We have presented results of quantum Berezinskii–Kosterlitz and Thouless transition for both the PT symmetry preserve and broken state for correlated many-body system. We have shown that a combination of spectral singularity and quantum criticality yields an exotic universality class which has no counterpart in known critical phenomena. We have found that the evidence of both hidden and conventional QBKT for the real part of the potential for the whole sets of RG equations and also for the conventional BKT equation, respectively. We have presented the exact solution for the second order RG flow lines for the both real and imaginary coupling. The topological excitations are different nature for the real and imaginary part of the potential. We have shown explicitly that for the PT symmetric phase, one can express the model Hamiltonian of the system in terms of effective single frequency-single field sine-Gordon field theory, whereas for the PT symmetry broken phase the effective Hamiltonian of the system is double frequency dual field sine-Gordon field theory. This work provides a new perspective for the study of PT symmetry quantum criticality.

## Methods

### Derivation of renormalization group equations

Our starting point is the sine-Gordan Hamiltonian

13 $$\begin{aligned} H = (\frac{{h} v}{2 \pi }) \int dx [ K {({\partial _x \theta (x)})}^2 + \frac{1}{K} {({\partial _x \phi (x)})}^2 ] + g_r \int dx cos (2 \phi (x)) + i g_i \int dx sin (2 \phi (x)) . \end{aligned}$$

Now we write the partition function $\mathscr {Z}$ in terms of fields as,

14 $$\begin{aligned} \mathscr {Z}= \int \mathscr {D}\phi \mathscr {D}\theta e^{-S_E[\phi ]}. \end{aligned}$$

where $S_E$ is the Euclidean action which can be written as $S_E= -\int dr \mathscr {L} = -\int dr (\mathscr {L}_0 + \mathscr {L}_{int})$, where $r=(\tau ,x)$. Thus the partition function is given by

15 $$\begin{aligned} \mathscr {Z}= & {} \int \mathscr {D}\phi \mathscr {D}\theta exp\left[ - \int _{-\Lambda }^{\Lambda } \frac{d\omega }{2\pi }|\omega | \left( \frac{|\phi (\omega )|^2}{2K} + \frac{K|\theta (\omega )|^2}{2}\right) \right. \nonumber \\&\left. - \int dr \left( g_r cos(2 \phi (x) ) + i g_i sin(2 \phi (x) ) \right) \right] . \end{aligned}$$

The first and second terms of the exponent of the above equation are $\mathscr {L}_0$ and $\mathscr {L}_{int}$, respectively.

Now we divide the fields into slow and fast modes and integrate out the fast modes. The filed $\phi$ is $\phi (r)=\phi _s(r)+\phi _f(r)$.

16 $$\begin{aligned} \phi _s(r)=\int _{-\Lambda /b}^{\Lambda /b} \frac{d\omega }{2\pi } e^{-i\omega r}\phi (\omega ) \;\;\;\; {\text{and}} \;\;\; \phi _f(r)=\int _{\Lambda /b<|\omega _n|<\Lambda } \frac{d\omega }{2\pi } e^{-i\omega r}\phi (\omega ). \end{aligned}$$

Here $\Lambda$ is the cut-off to start with and *b* is factor greater than one. It is clear from the above definition of faster and slower mode. One can make the average over the fast mode in order to get an effective action for the slower mode. Thus $\mathscr {Z}$ is

17 $$\begin{aligned} \mathscr {Z}=\int \mathscr {D}\phi _s \mathscr {D}\phi _f e^{-S_f(\phi _f)} e^{-S_{int}(\phi )}. \end{aligned}$$

Using the relation $\left\langle A \right\rangle _f = \int \mathscr {D}\phi _f e^{-S_f(\phi _f)} A$, one can write

18 $$\begin{aligned} \mathscr {Z}=\int \mathscr {D}\phi _s e^{-S_s(\phi _s)} \left\langle e^{-S_{int}(\phi )} \right\rangle _f. \end{aligned}$$

We write the effective action as

19 $$\begin{aligned} e^{-S_{eff}(\phi _s)}=e^{-S_s(\phi _s)} \left\langle e^{-S_{int}(\phi )} \right\rangle _f. \end{aligned}$$

Taking $\ln$ on both sides give, $S_{eff}(\phi _s)= S_s(\phi _s) - \ln \left\langle e^{-S_{int}(\phi )} \right\rangle _f.$ By writing the cumulant expansion up to third order, we have

20 $$\begin{aligned} S_{eff}(\phi _s)= & {} S_s(\phi _s)+ \left\langle S_{int}(\phi ) \right\rangle _f - \frac{1}{2} \left( \left\langle S^2_{int}(\phi ) \right\rangle _f - \left\langle S_{int}(\phi ) \right\rangle ^2_f \right) + \frac{1}{6} \left( \left\langle S^3_{int}(\phi ) \right\rangle _f - 3 \left\langle S^2_{int}(\phi ) \right\rangle _f \left\langle S_{int}(\phi ) \right\rangle _f + 2 \left\langle S_{int}(\phi ) \right\rangle ^3_f \right) . \end{aligned}$$

21 $$\begin{aligned} \left\langle S_{int}(\phi )\right\rangle= & {} \int dr \left[ g_r \left\langle \cos \left( 2 \phi (r) \right) \right\rangle _f \right] + \left[ i g_i \left\langle \sin \left( 2 \phi (r) \right) \right\rangle _f \right] \end{aligned}$$

22 $$\begin{aligned} \boxed {\int dr \left[ g_r \left\langle \cos \left( 2 \sqrt{\pi } \theta (r)\right) \right\rangle _f \right] = b^{-{K}} \int dr \left[ g_r \cos \left( 2 \sqrt{\pi } \theta _s(r)\right) \right] } \end{aligned}$$

23 $$\begin{aligned} \boxed {\int dr \left[ g_i \left\langle \cos \left( 2 \sqrt{\pi } \theta (r)\right) \right\rangle _f \right] = b^{-{K}} \int dr \left[ g_i \cos \left( 2 \sqrt{\pi } \theta _s(r)\right) \right] } \end{aligned}$$

Similarly, Now we calculate the second order cumulant term of the action $S_{eff} (\phi )$

24 $$\begin{aligned} -\frac{1}{2}(\left\langle S_{int}^2\right\rangle -\left\langle S_{int}\right\rangle ^2) = -\frac{1}{2} \int dr dr^{\prime } \left\{ {g_r}^2 [....] - {g_i}^2 [....] + i g_r g_i [....] + i g_i g_r [....] \right\} , \end{aligned}$$

where the dotted term represents the expectation value of the correlation function of sine-Gordon operators, which we evaluate below.

25 $$\begin{aligned} -\frac{{g_r}^2}{2} \int dr dr^{\prime } \left\{ \left\langle \cos [2 \phi (r)] \cos [2 \phi (r^{\prime })] \right\rangle - \left\langle \cos [2 \phi (r)] \right\rangle \left\langle \cos [2 \phi (r^{\prime })] \right\rangle \right\} = \frac{{g_r}^2}{4} \left( 1-b^{-{2}{K}} \right) \int dr (\partial _r \phi _s(r))^2. \end{aligned}$$

Similarly,

26 $$\begin{aligned} \frac{{g_i}^2}{2} \int dr dr^{\prime } \left\{ \left\langle \sin [2 \phi (r)] \cos [2 \phi (r^{\prime })] \right\rangle - \left\langle \sin [2 \phi (r)] \right\rangle \left\langle \sin [2 \phi (r^{\prime })] \right\rangle \right\} = - \frac{{g_i}^2}{4} \left( 1-b^{-2K} \right) \int dr (\partial _r \phi _s(r))^2. \end{aligned}$$

We obtain the following relation by comparison of rescaled $g_r$ term (Eqs. 22,  23) using the rescaled relation as $b = e^{dl}$.

27 $$\begin{aligned} \bar{g_r}= & {} {g_r} b^{(2-{K})} \,\,\,\,\,\,\,\,\,\,\,\rightarrow \boxed {\frac{d g_r}{dl}= \left( 2-{K}\right) \Delta } . \end{aligned}$$

28 $$\begin{aligned} \bar{g_i}= & {} g_i b^{(2-K)} \,\,\,\,\,\,\,\,\,\,\,\,\rightarrow \boxed {\frac{dg_i}{dl}= \left( 2-K\right) g_i } . \end{aligned}$$

Comparison of rescaled *K* terms from the contribution of $\phi$ (Eqs. 25, 13), gives,

29 $$\begin{aligned} \frac{1}{\bar{K}}= \frac{1}{K} + \frac{{g_r}^2}{2 K}\left( b^2 - b^{(2-K)}\right) \end{aligned}$$

$$b = e^{dl}$$

30 $$\begin{aligned} \frac{1}{\bar{K}}= \frac{1}{K} + {{g_r}^2} dl \,\,\,\,\,\,\,\,\,\,\,\rightarrow \frac{dK}{dl}= -{{g_r}^2}. \end{aligned}$$

Similarly one can find the analytical expression for $g_i$.

31 $$\begin{aligned} \frac{dK}{dl}= {{g_i}^2}. \end{aligned}$$

We obtain the final form of $\frac{dK}{dl}$ after the combination of the above two contribution from $\phi$ and $\theta$ for *K*.

32 $$\begin{aligned} \frac{dK}{dl}= ( {{g_i}^2}- {{g_r}^2 } ) K^2 . \end{aligned}$$

Thus finally we obtain the 2nd order RG equations which are the following

33 $$\begin{aligned} \frac{d g_r }{dl}&= \left( 2-K \right) g_r \nonumber \\ \frac{d g_i }{dl}&= \left( 2-K \right) g_i \nonumber \\ \frac{dK}{dl}&= ({g_i}^2 - {g_r}^2 ) K^2 . \end{aligned}$$

These 2nd order RG equations are consistent, if your take the limit of 2nd order of Ref.^29^, it is the same RG equations.

The 2nd order QBKT equations (Eqs. 7, 8) for the coupling $g_r$ and $g_i$ can be obtained by taking the limits $g_i =0$ and $g_r =0$ respectively.

Now we calculate the third order terms of cumulant expansion for the effective action (Eq. 20),

34 $$\begin{aligned}&\frac{1}{6}\left( \left\langle S_{int}^3 \right\rangle -3\left\langle S_{int}^2 \right\rangle \left\langle S_{int} \right\rangle +2 \left\langle S_{int} \right\rangle ^3\right) = \frac{1}{6} \int d\tau d\tau ^{\prime } d\tau ^{\prime \prime }\nonumber \\&\quad \times \left\{ {g_r}^3 \left[ ..... \right] - i {g_i}^3 \left[ ..... \right] + i {g_r}^2 {g_i} \left[ ..... \right] - g_r {g_i}^2 \left[ ..... \right] \right\} \end{aligned}$$

Now we follow the same analytical calculations what we have done during the derivation of second order RG equations and finally obtain these RG equations for the third order RG process which is consistent with Ref.^29^.

35 $$\begin{aligned} \frac{d g_r }{dl}&= \left( 2-K \right) g_r + 5 {g_r}^3 - 5 {g_i}^2 g_r \nonumber \\ \frac{d g_i }{dl}&= \left( 2-K \right) g_i - 5 {g_i}^3 + 5 {g_r}^2 g_i \nonumber \\ \frac{dK}{dl}&= ({g_i}^2 - {g_r}^2) K^2 . \end{aligned}$$

The 3rd order QBKT equations (Eqs. 9,  10) for the coupling $g_r$ and $g_i$ can be obtained by taking the limits $g_i =0$ and $g_r =0$ respectively.
